# A review of the genus *Lordiphosa* Basden in India, with descriptions of four new species from the Himalayan region (Diptera, Drosophilidae)

**DOI:** 10.3897/zookeys.688.12590

**Published:** 2017-08-08

**Authors:** Rajendra S. Fartyal, Pradeep C. Sati, Sushmika Pradhan, Mukul C. Kandpal, Masanori J. Toda, Rabindra N. Chatterjee, Birendra K. Singh, Asha Bhardwai

**Affiliations:** 1 Systematics, Cytogenetics and Molecular Laboratory, Department of Zoology and Biotechnology, Srinagar-Garhwal, Uttarakhand, India; 2 P.G. Department of Zoology, Darjeeling Government College, Darjeeling, West Bengal, India; 3 Genetics Research Unit, Department of Zoology, University of Calcutta, West Bengal, India; 4 Cytogenetics Laboratory, Department of Zoology, Kumaun University, Nainital, Uttarakhand, India; 5 Hokkaido University Museum, Hokkaido University, N10, W8, Kita-ku, Sapporo 060-0810, Japan

**Keywords:** Darjeeling, key, *Lordiphosa denticeps* species group, *Lordiphosa nigricolor* species group, new synonymy, redescription, Uttarakhand

## Abstract

All Indian species of the genus *Lordiphosa* Basden are reviewed, with descriptions of four new species, *L. curva* Fartyal & Toda, **sp. n**. of the *denticeps* species group and *L. ayarpathaensis* Kandpal & Singh, **sp. n.**, *L. makaibarensis* Pradhan & Chatterjee, **sp. n.** and *L. srinagarensis* Sati & Fartyal, **sp. n.** of the *nigricolor* species group. Two of the new species, *L. ayarpathaensis* and *L. makaibarensis*, were found visiting flowers of *Hedychium spicatum* and *Datura suaveolens*, respectively. This is the first record of flower visitation in *Lordiphosa* flies. In addition, *L. parantillaria* (Kumar & Gupta, 1990), **syn. n.** is synonymized with *L. antillaria* (Okada, 1984). Supplementary and revised descriptions for *L. antillaria* and *L. neokurokawai* (Singh & Gupta, 1981) and a key to all Indian species of *Lordiphosa* are provided.

## Introduction

The genus *Lordiphosa* Basden is a moderately-sized genus of the family Drosophilidae, currently comprising 57 species (Brake and Bächli 2008). The taxonomy of this genus had once been confused by assignment of some species to the subgenera *Sophophora* Sturtevant (Kikkawa and Peng 1938, Okada 1956, 1966, 1974, 1977, Lee 1959, Takada and Okada 1960, Bock and Wheeler 1972), *Hirtodrosophila* Duda (Okada and Sasakawa 1956, Okada 1966, 1967, 1971, 1988, Singh and Gupta 1981), or *Drosophila* Fallén (Duda 1935) of the genus *Drosophila*. However, some revisional works (Laštovka and Máca 1978, Okada 1984, 1990) reclassified those species into the subgenus Lordiphosa of the genus *Drosophila*. Grimaldi (1990) elevated *Lordiphosa* to the generic rank according to morphological characters. Furthermore, Hu and Toda (2001) showed, by a cladistic analysis focusing on *Lordiphosa*, that the *tenuicauda* species group, initially included in *Lordiphosa* (Toda 1983, Hu et al. 1999), is remotely related to the *Lordiphosa* proper, and Hu and Toda (2002) transferred all species of the *tenuicauda* group to the revised genus *Dichaetophora* Duda. A molecular phylogenetic study by Gao et al. (2011) has revealed that *Lordiphosa* is the sister group to the Neotropical *Sophophora* consisting of the *Drosophila saltans* and *D. willistoni* species groups.

The genus *Lordiphosa* is distributed from the tropics of Oriental Region (Okada 1984, 1988, Toda unpublished data) to the subarctics of Palearctic Region (Toda et al. 1996, Bächli et al. 2004), with the highest species richness in the subtropics of Oriental Region (Okada 1966, 1984, Zhang 1993a, b, Zhang and Liang 1992, 1994, Quan and Zhang 2001, 2003). However, this genus has been poorly represented in India: only seven species have been recorded (Dwivedi and Gupta 1980, Singh and Gupta 1981, Kumar and Gupta 1990, Gupta and Gupta 1991, De and Gupta 1996, Gupta 2005, Upadhyay and Singh 2007).

Four new species of *Lordiphosa* have been discovered from India. Two of them were found visiting flowers of *Hedychium spicatum* Smith (Zingiberaceae) in Kumaon, Uttarakhand and of *Datura suaveolens* (Humb. & Bonpl. exWilld.) Bercht. & J. Presl (Solanaceae) in Darjeeling, West Bengal. Until now, *Lordiphosa* flies were known for breeding on herbage plants, and their larvae feeding on decayed tissues of leaves and stems (Kimura et al. 1977, Shorrocks 1982, Toda et al. 1984) or living tissue of leaves as leaf minors (Okada and Sasakawa 1956). This paper describes the four new species, and reviews all known Indian species of *Lordiphosa* with supplementary and revised descriptions for some species, and provides a key to all Indian species of *Lordiphosa*.

## Materials and methods

Specimens used for the present study were collected from four different hill stations of the Himalayan region in India: Chopta (2,700 m a.s.l.; 30°29'N, 79°10'E) in Rudraprayag district; Ayarpatha (2,278 m a.s.l.; 29°23'N, 79°27'E) in Nainital district; Kurseong subdivision of Darjeeling hills (1,458 m a.s.l.; 26°53'N, 88°17'E) in West Bengal; and HNBGU Forestry nursery (560 m a.s.l.; 30°13'N, 78°47'E) at Chauras Srinagar Garhwal in Uttarakhand. These localities are covered with dense mixed-deciduous subtropical forests, under extremely moist condition due to heavy rainfall during the summer monsoon season. The temperature ranges approximately from 3°C to 24°C. Specimens were collected by net sweeping or directly from flowers of *Hedychium spicatum* and *Datura suaveolens* by an aspirator, and preserved in 70% ethanol. In addition, some specimens collected from China were examined to give supplementary and revised descriptions for some known species.

External morphology of adult flies was examined under a stereomicroscope and metric characters were measured with an ocular micrometer. To observe detailed structures, the male and female terminalia and some other organs were detached from the body, cleared by warming in 10% KOH solution at approximately 100°C for several minutes, mounted in a droplet of glycerin on a cavity slide, examined under a light microscope, and some samples imaged using a DinoLite® Digital Eyepiece Camera.

The morphological terminology and the definition of measurements and indices mostly follow McAlpine (1981), Zhang and Toda (1992) and Hu and Toda (2001). All the holotypes and some paratypes of new species are deposited in the Department of Zoology, H.N.B Garhwal University, Chauras Campus, Srinagar-Garhwal, Uttarakhand, India (**DZHNBGU**), some paratypes in Museum of Zoological Survey of India, Kolkata, India (**MZSIK**) and the remaining paratypes in the Systematic Entomology, Hokkaido University Museum, Hokkaido University, Sapporo, Japan (**SEHU**).

## Systematic accounts

### Genus Lordiphosa

Taxon classificationAnimaliaDipteraDrosophilidae

Basden

Lordiphosa Basden, 1961: 186 (as a subgenus of Drosophila); Laštovka and Máca 1978: 404; Okada 1984: 571. Type species: Drosophila fenestrarum Fallén, 1823.
Lordiphosa : Grimaldi, 1990: 121 (new status as genus); De and Gupta 1996: 131; Bächli et al. 2004: 250.

#### Diagnosis.

**Figure 1.**
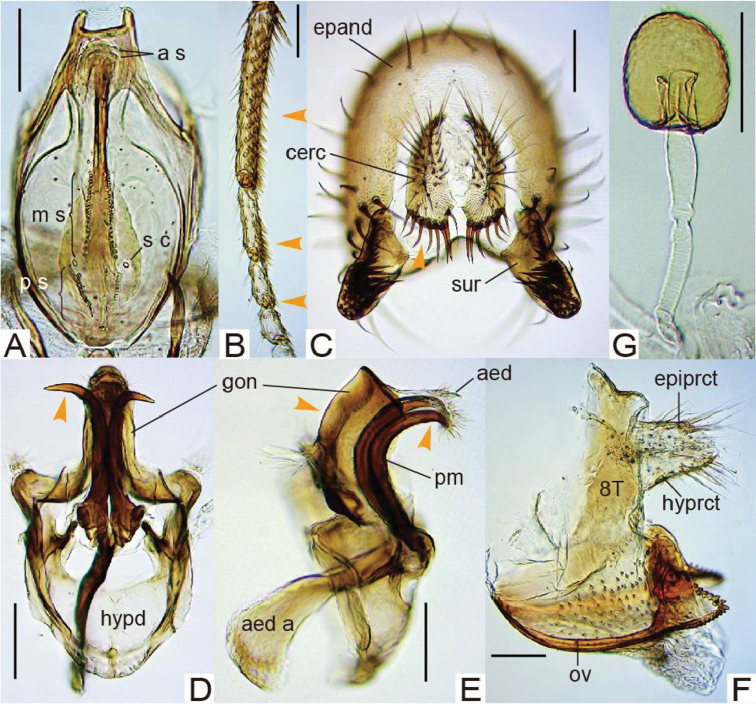
*Lordiphosa neokurokawai* (Singh & Gupta, 1981) (♂♀ from Kunming, Yunnan, China): **A** cibarium: anterior sensilla (a s), medial sensilla (m s), posterior sensilla (p s) and sensilla campaniformia (s c) (dorsal view) **B** foreleg tarsus **C** periphallic organs: epandrium (epand), cercus (cerc) and surstylus (sur) (caudal view) **D, E** phallic organs: aedeagus (aed), aedeagal apodeme (aed a), gonopods (gon), hypandrium (hypd) and paramere (pm) (**D** ventral view **E** lateral view) **F** ♀ terminalia: tergite VIII (8T), epiproct (epiprct), hypoproct (hyprct) and oviscapt (ov) **G** spermatheca. Arrowheads indicate the diagnostic characters. Scale bars 0.1 mm.

**Figure 2.**
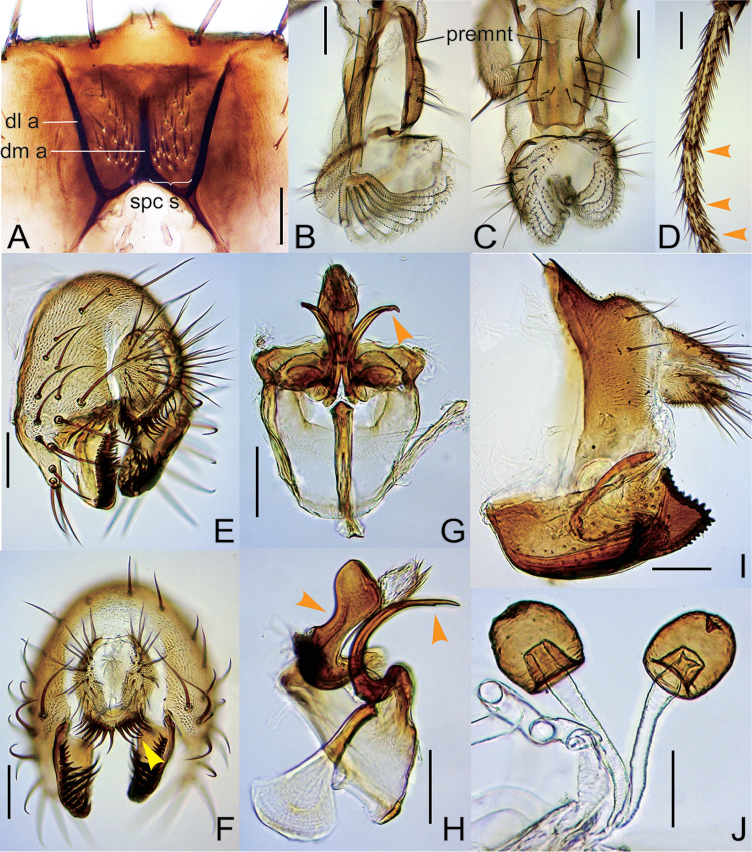
*Lordiphosa curva* Fartyal & Toda, sp. n. (♂♀ paratypes from Chopta Forest, Uttarakhand, India): **A** occiput: dorsolateral arms (dl a) and dorsomedial arm (dm a) of tentorial apodeme and supracervical setae (spc s) **B, C** proboscis: prementum (premnt) (**B** lateral view **C** posterior view) **D** foreleg tarsus **E, F** periphallic organs (**E** caudolateral view **F** caudal view) **G, H** phallic organs (**G** ventral view **H** lateral view) **I** ♀ terminalia **J** spermathecae. Scale bars 0.1 mm.

**Figure 3.**
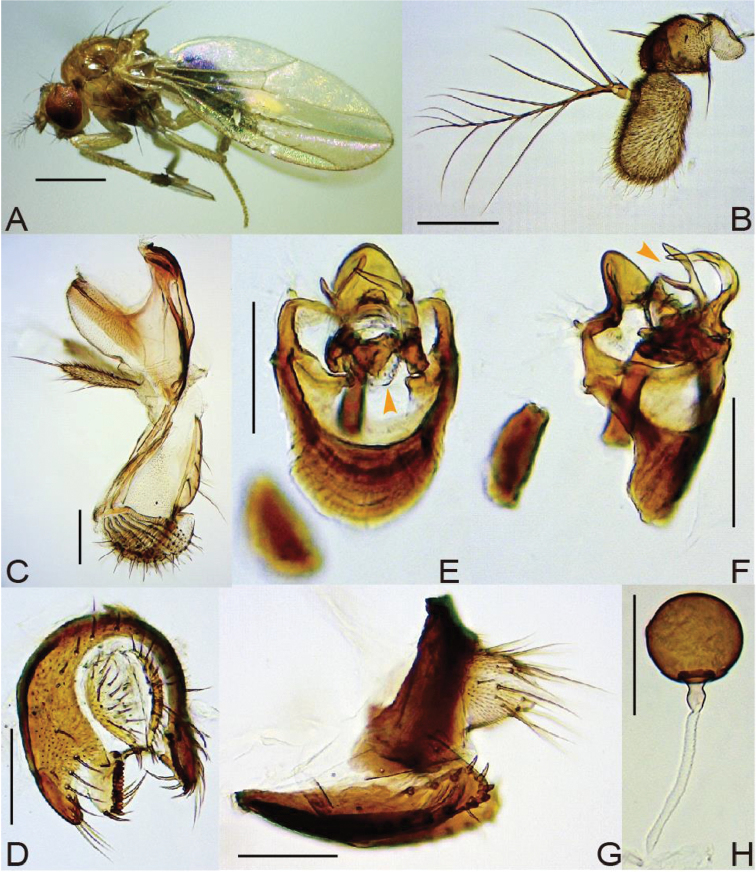
*Lordiphosa antillaria* (Okada, 1984) (♂♀ from Bagdogra, Assam, West Bengal, India): **A** ♂ left lateral habitus **B** antenna **C** mouthparts (lateral view) **D** periphallic organs (caudolateral view) **E, F** phallic organs (aedeagal apodeme broken) (**E** ventral view **F** ventrolateral view) **G** ♀ terminalia **H** spermatheca. Scale bars 0.5 mm **A**; 0.1 mm **B–H**.

**Figure 4.**
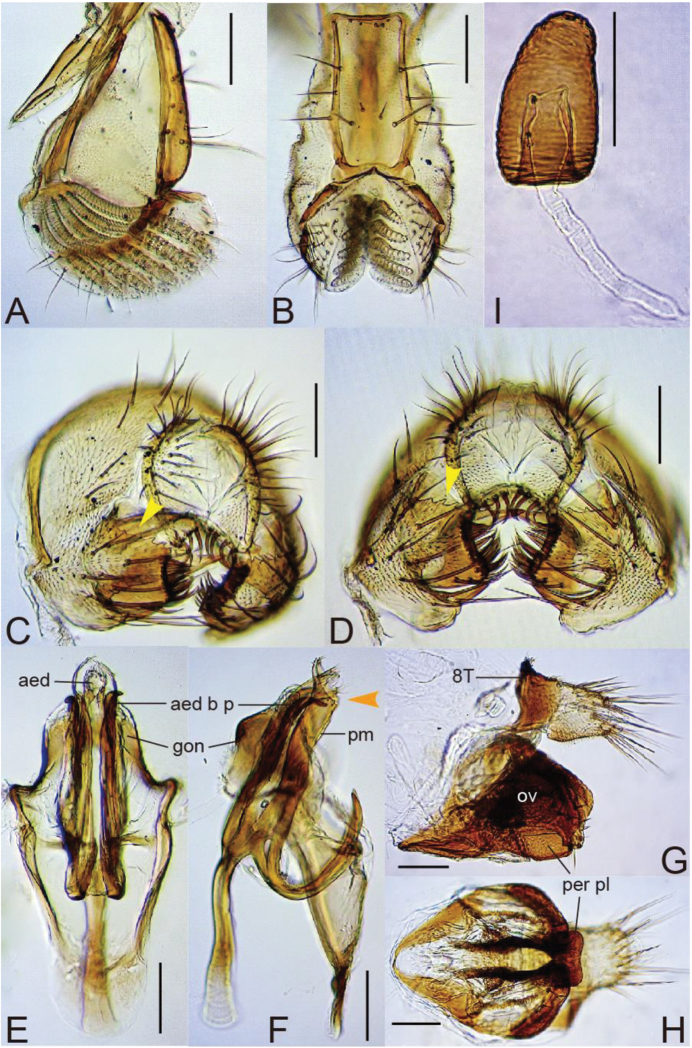
*Lordiphosa ayarpathaensis* Kandpal & Singh, sp. n. (♂♀ paratypes from Ayarpatha, Nainital district, Kumaon, Uttarakhand, India): **A, B** proboscis (**A** lateral view **B** posterior view) **C, D** periphallic organs (**C** caudolateral view **D** caudoventral view) **E, F** phallic organs: aedeagal basal processes (aed b p) (**E** ventral view **F** lateral view) **G, H** ♀ terminalia: perineal plate (per pl) (**G** lateral view **H** ventral view) **I** spermatheca. Scale bars 0.1 mm.

Prementum thicker in ventral than in dorsal portion from lateral view (Figs 2B, 3C, 4A), ventrally more or less expanded in posterior view (Figs 2C, 4B). Paramere ventrobasally articulated with hypandrium (Figs 1D, 2H, 3E, 4E, F, 7D). Hypandrium lacking paramedian setae.

### Lordiphosa denticeps

Taxon classificationAnimaliaDipteraDrosophilidae

species group

Drosophila (Hirtodrosophila) denticeps species-group, Okada, 1967: 3.
Lordiphosa denticeps species-group: Zhang, 1993b: 144.

#### Diagnosis.

Male foreleg tarsomeres I to III often with sex combs (Figs 1B, 2D). Surstylus with numerous recurved setae on ventral portion of inner surface in addition to primary prensisetae on caudobasal margin (Figs 1C, 2F). Ventral margin of cercus tapering or truncated, fringed with a row of stout spines (Figs 1C, 2F). Aedeagus membranous, hirsute apically, fused with posterior, roof-like gonopod (Figs 1E, 2H). Oviscapt with numerous lateral ovisensilla (Figs 1F, 2I).

#### Remarks.

Okada (1967) proposed the *denticeps* group as a new species group of *Hirtodrosophila* (a subgenus of *Drosophila* at that time), including two species so far described, *denticeps* Okada & Sasakawa, 1956 and *tripartita* Okada, 1966. However, it had been noticed that these two species have aberrant morphological characters inconsistent with the definition of *Hirtodrosophila*. Three more species were subsequently added to this species group (Okada 1971, Singh and Gupta 1981). Then Okada (1990) transferred the members of this species group to *Lordiphosa* (a subgenus of *Drosophila* at that time), but considered that the *denticeps* group was synonymous with the *nigricolor* group proposed by Laštovka and Máca (1978). Then, Grimaldi (1990) elevated *Lordiphosa* to the generic rank, and Zhang (1993b) resurrected and redefined the *denticeps* group as a species group independent from the *nigricolor* group in the genus *Lordiphosa*.

#### Key to Indian species of the *denticeps* group

**Table:** 

1	Arista with one ventral branch (except terminal fork)	**2**
–	Arista with two ventral branches	***tripartita* (Okada, 1966)**
2	Setae of all, approximately 15 TBRs (Transverse Bristle Rows; Baumina and Kopp 2007) on tarsomere I of ♂ foreleg thick, forming sex combs (Fig. 1B); gonopod not concaved proximally on posterior margin in lateral view (Fig. 1E); ♀ abdominal tergite VIII without setae (Fig. 1F); oviscapt with approximately 100 lateral ovisensilla (Fig. 1F); spermathecal capsule apically not indented (Fig. 1G)	***neokurokawai* (Singh & Gupta, 1981)**
–	Setae of only distal most TBR on tarsomere I of ♂ foreleg thick, forming sex comb (Fig. 2D); gonopod concaved proximally on posterior margin in lateral view (Fig. 2H); ♀ abdominal tergite VIII dorsally with approximately two setae per side (Fig. 2I); oviscapt with approximately 35 lateral ovisensilla (Fig. 2I); spermathecal capsule apically indented (Fig. 2J)	***curva* Fartyal & Toda, sp. n.**

### Lordiphosa neokurokawai

Taxon classificationAnimaliaDipteraDrosophilidae

(Singh & Gupta)

Fig. 1

Drosophila (Hirtodrosophila) neokurokawai Singh & Gupta, 1981: 207.
Lordiphosa neokurokawai : Zhang, 1993b: 145.

#### Specimens examined.

CHINA: 1♂, Sichuan, Mt. Emei, 2,000 m a.s.l., 19 July 1992; 1♂, 1♀, Yunnan, Kunming, 22 March 2005 (all in SEHU).

#### Diagnosis.

Sex combs composed of thick setae of all, approximately 15 TBRs on anteroventral surface of tarsomere I, of four distal TBRs on tarsomere II and of two distal TBRs on tarsomere III (Fig. 1B). Cercus ventrally broadest, nearly horizontally truncated, with approximately seven large, stout spines on ventral margin (Fig. 1C). Gonopod not concaved proximally on posterior margin in lateral view (Fig. 1E). Paramere basally with strong knob, subapically without spinule (Fig. 1D, E). Female abdominal tergite VIII without setae (Fig. 1F). Oviscapt with approximately 100 small, trichoid lateral ovisensilla (Fig. 1F). Spermathecal capsule apically not indented (Fig. 1G).

#### Description

(supplementary and revised). **Adult male.** *Head*. Eye with interfacetal setulae. Approximately 15 supracervical setae thin, apically more or less curved and pointed; postocular setae approximately 18; occipital setae 30–31, including medial tiny ones. Dorsolateral arms of tentorial apodeme divergent, nearly straight, reaching to fronto-orbital plate; dorsomedial arm 1/3 as long as dorsolateral arm. Interspace between antennal sockets narrower than half of socket width; first flagellomere with only one small invaginated pouch (“sacculus” called by earlier taxonomists: Ferris 1965); arista with 4–5 dorsal and one ventral branches in addition to terminal fork. Facial carina slightly elevated, narrower and shorter than first flagellomere, without setulae below. Subvibrissal seta distinctly shorter than vibrissa; additional row of oral setulae present above marginal row on anterior portion. Palpus with one prominent terminal and several short, subapical to lateroventral setae, without setulae on basal lobe. Cibarium (Fig. 1A) thickened on anterior margin, not dilated laterad in anterior portion; anterolateral projections shorter than half width of anterior margin; dorsal sclerite pear-shaped in dorsal view, anteriorly convex in lateral view; anterior sensilla two pairs, widely arranged in square behind anterior margin of hypopharynx; 23–26 medial sensilla arranged in anteriorly slightly convergent rows; two sensilla campaniformia; posterior sensilla very long, trichoid, gently curved forward, approximately 17 arranged in anteriorly divergent rows; somewhat sclerotized, thickened (in lateral view), anterior portion of hypopharynx shorter than 1/5 length of cibarium. Prementum ventrally slightly expanded. Labellum with five pseudotracheae per side.

*Thorax*. Postpronotal lobe with two prominent setae. Posterior dorsocentral seta situated nearer to anterior margin of scutellum than to anterior dorsocentral seta. Prescutellar setae absent. One or a few acrostichal setulae in lines with and anterior to dorsocentral setae thicker and longer than others. Mid katepisternal seta shorter than anterior katepisternal seta; anterior katepisternal seta thicker than aristal branches; no setula present anteriorly to anterior katepisternal seta.

*Wing* hyaline. Veins light brown; crossveins not clouded; bm-cu crossvein absent; R_2+3_ nearly straight; R_4+5_ and M_1_ nearly parallel. Two C_1_ setae unequal in size.

*Legs*. Foreleg tarsus with neither tuft of dense, soft hairs nor long setae. Foreleg tarsomere I as long as three succeeding tarsomeres together; midleg one slightly longer than three succeedings together; hindleg one slightly longer than rest together.

*Abdomen*. Setigerous sternite VI present.

*Terminalia* (Fig. 1C–E). Epandrium shallowly and widely notched on posterior mid-dorsal margin (Fig. 1C), nearly entirely pubescent except antero-lateral to -ventral margin, gently curved on caudosubmedial margin near articulation to surstylus, ventrally narrowing but apically somewhat roundish and not sclerotized, with approximately16 setae on medial to dorsal portion, approximately six setae on ventral lobe and unpubescent, inward fold on caudoventral margin. Surstylus articulated to epandrium, simple plate longer than wide and apically round, with 8–9 prensisetae on caudodorsal margin and 42–43 recurved setae on ventral portion of inner surface but neither pubescence nor peg-like setae on outer surface (Fig. 1C). Cercus separated from epandrium, nearly entirely pubescent except for lateral margin, with 32–33 setae (Fig. 1C). Membrane between cercus and epandrium pubescent dorsally. Lateral lobe of tenth sternite larger than median lobe. Hypandrium anteriorly fringed with arched apodeme, slightly pubescent on caudolateral plates fused to gonopod, with a pair of narrow sclerotized processes connecting between bases of parameres and lateral margins of hypandrium (Fig. 1D). Paramere long, sclerotized process curved ventrad medially and outward apically (Fig. 1D, E). Aedeagal basal processes degenerated. Gonopods fused with each other, forming roof-like plate posteriorly surrounding aedeagus (Fig. 1E).

**Adult female.** Head, thorax, wings and legs as in male, except for absence of sex combs on foreleg tarsus.

*Terminalia* (Fig. 1F, G). Tergite VIII entirely narrow, pubescent only on posterior portion (Fig. 1F). Epiproct and hypoproct entirely pubescent and setigerous (Fig. 1F). Oviscapt subapically broadest, apically triangular, with approximately 13 and 9 apically blunt, peg-like ovisensilla on apico-ventral and -dorsal margins, respectively; subapical, trichoid ovisensillum as long as largest, marginal one (Fig. 1F). Spermathecal capsule ellipsoidal; introvert half as deep as capsule height; outer duct not wrinkled in distal 1/3 (Fig. 1G).

#### Distribution.

Southwestern China (Sichuan*, Yunnan), India (West Bengal) [* new record].

#### Remarks.

This species was first described by Singh and Gupta (1981) based on three male specimens collected from Darjeeling, West Bengal, India. Later, Zhang (1993b) reported this species from southwestern China, based on some male and female specimens collected from Kunming, Yunnan, but did not describe the female characters. Here, the description of the female is provided, with a supplementary and revised description for male based on the specimens collected from southwestern China.

### Lordiphosa curva

Taxon classificationAnimaliaDipteraDrosophilidae

Fartyal & Toda sp. n.

http://zoobank.org/F7A1A1E5-DBE8-4D82-879A-112056A9710E

Fig. 2

#### Type material.

*Holotype*. ♂: INDIA: Uttarakhand, Rudraprayag District, Chopta Forest. 30°27.560'N, 79°15.234'E, 2,700 m a.s.l., 31 August 2010, R. S. Fartyal leg. (DZHNBGU).

*Paratypes*. INDIA: 1♂, 1♀, same data as the holotype except 1 September 2010 (MZSIK, SEHU).

#### Diagnosis.

Sex combs composed of thick setae of only distal most TBR on tarsomeres I–III (Fig. 2D). Cercus ventrally somewhat obliquely truncated, with approximately eight large, stout spines on ventral margin (Fig. 2F). Gonopod concaved proximally on posterior margin in lateral view (Fig. 2H). Paramere basally curved ventrad, apically much narrow, subapically with spinule (Fig. 2G, H). Female abdominal tergite VIII dorsally with approximately two setae (Fig. 2I). Oviscapt with approximately 35 small, lateral ovisensilla (Fig. 2I). Spermathecal capsule apically indented (Fig. 2J).

#### Description

(not referring to characters commonly seen in the foregoing species, *L. neokurokawai*). **Adult male.** *Head*. Eye with dense, interfacetal setulae. Supracervical setae 16–18 (Fig. 2A); postocular setae approximately 19; occipital setae 21–25. Dorsolateral arms of tentorial apodeme divergent, apically curved outward; dorsomedial arm half as long as dorsolateral arm (Fig. 2A). Occiput orange yellow, medially dark brown; ocellar triangle and fronto-orbital plates glossy, orange yellow; frontal vittae mat, greyish orange. Pedicel greyish orange yellow; first flagellomere grey; arista with 3–4 dorsal and one ventral branches in addition to terminal fork. Face orange yellow. Gena orange yellow but dark brown on anteroventral margin. Clypeus orange brown. Palpus yellow. Cibarium: anterolateral projections longer than half width of anterior margin; medial sensilla 20–21; posterior sensilla approximately 20. Prementum ventrally slightly expanded, thicker in ventral than in dorsal portion from lateral view (Fig. 2B, C). Labellum with five pseudotracheae per side (Fig. 2B).

*Thorax*. Postpronotal lobe grey yellow, with two prominent setae: upper one 0.8 as long as lower one. Scutum and scutellum grey yellow but grey brown medially. Thoracic pleura grey yellow, with dark grey patches. Acrostichal setulae in six rows. Basal scutellar setae divergent; apicals cruciate.

*Wing*. C_1_ setae two, subequal in size. Halter opaque white.

*Legs* grey yellow; tarsomere Vs of all legs darker. Foreleg femur with 4–6 long setae in two rows on outer side. Fore- and mid-leg tarsomere Is longer than three succeeding tarsomeres together; hindleg one longer than rest together. Preapical, dorsal setae present on tibiae of all legs; apical setae on tibiae of fore- and mid-legs.

*Abdomen*. Tergites grey yellow, each posteriorly darker. Sternites yellow.

*Terminalia* (Fig. 2E–H). Epandrium with 11–14 setae on medial to dorsal portion and approximately four setae on ventral lobe (Fig. 2E). Surstylus with 6–7 apically pointed prensisetae arranged along caudobasal margin and 40–42 recurved setae on ventral portion of inner surface (Fig. 2E, F). Cercus nearly entirely pubescent except for lateral to ventral margin, with 21–24 setae (Fig. 2E, F). Hypandrium caudolaterally pubescent and fused to gonopod, with a pair of inward extended plates apically articulated to bases of parameres (Fig. 2G).

*Measurements* (holotype / 1♂ paratype, in mm). BL (straight distance from anterior edge of pedicel to tip of abdomen) = 2.65 / 2.77, ThL (distance from anterior notal margin to apex of scutellum) = 1.30 / 1.42, WL (distance from humeral cross vein to wing apex) = 3.47 / 3.67, WW (maximum wing width) = 1.40 / 1.54.

*Indices* (holotype / 1♂ paratype, in ratio). FW/HW (frontal width / head width) = 0.51 / 0.55, ch/o (maximum width of gena / maximum diameter of eye) = 0.28 / 0.27, prob (proclinate orbital seta / posterior reclinate orbital seta in length) = 0.70 / 0.81, rcorb (anterior reclinate orbital seta / posterior reclinate orbital seta in length) = 0.32 / 0.35, vb (subvibrissal seta / vibrissa in length) = 0.52 / 0.50, dcl (anterior dorsocentral seta / posterior dorsocentral seta in length) = 0.65 / 0.64, sctl (basal scutellar seta / apical scutellar seta in length) = 1.18 / 1.17, sterno (anterior katepisternal seta / posterior katepisternal seta in length) = 0.57 / 0.50, orbito (distance between proclinate and posterior reclinate orbital setae / distance between inner vertical and posterior reclinate orbital setae) = 0.54 / 0.53, dcp (distance between ipsilateral dorsocentral setae / distance between anterior dorsocentral setae) = 0.55 / 0.57, sctlp (distance between ipsilateral scutellar setae / distance between apical scutellar setae) = 1.09 /1.03, C (2nd costal section between subcostal break and R_2+3_ / 3rd costal section between R_2+3_ and R_4+5_) =3.70 / 3.21, 4c (3rd costal section between R_2+3_ and R_4+5_ / M_1_ between r-m and dm-cu) = 0.62 / 0.69, 4v (M_1_ between dm-cu and wing margin / M_1_ between r-m and dm-cu) = 1.62 / 1.62, 5x (CuA_1_ between dm-cu and wing margin / dm-cu between M_1_ and CuA_1_) = 1.64 / 1.70, ac (3rd costal section between R_2+3_ and R_4+5_ / distance between distal ends of R_4+5_ and M_1_) = 2.08 / 2.51, M (CuA_1_ between dm-cu and wing margin / M_1_ between r-m and dm-cu) = 0.49 / 0.50, C3F (length of heavy setation in 3rd costal section + length of light setation in 3rd costal section) = 0.50 / 0.58.

**Adult female.** Head, thorax, wings, and legs as in male, except for absence of sex combs on foreleg tarsus.

*Terminalia* (Fig. 2I, J). Tergite VIII pubescent laterally to dorsally (Fig. 2I). Epiproct and hypoproct entirely pubescent and setigerous (Fig. 2I). Oviscapt broad from basal to subapical portion, apically triangular, with 9–11 apically blunt, stout, peg-like ovisensilla on apicodorsal margin and 13–15 ones proximally reducing in size and increasing in interspace on ventral margin (Fig. 2I). Spermathecal capsule ellipsoidal, basally horizontally wrinkled; introvert 2/5 as deep as capsule height (Fig. 2J).

*Measurements* (1♀ paratype, in mm). BL = 2.84, ThL = 1.39, WL = 3.54, WW = 1.44.

*Indices* (1♀ paratype, in ratio). FW/HW = 0.50, ch/o = 0.28, prorb = 0.76, rcorb = 0.34, vb = 0.38, dcl = 0.66, sctl = 1.26, sterno = 0.48, orbito = 0.57, dcp = 0.54, sctlp = 1.20, C = 3.30, 4c = 0.69, 4v = 1.64, 5x = 1.66, ac = 2.64, M = 0.50, C3F = 0.46.

#### Etymology.

The specific name curva = “curved” refers to the paramere basally curved ventrally.

#### Distribution.

India (Uttarakhand).

#### Remarks.

This species closely resembles *L. neokurokawai* in having only one ventral branch of arista, the long, apically pointed paramere curved ventrad medially and outward apically, and the epandrium and the surstylus nearly identical in morphology, but can be clearly distinguished from it by the diagnostic characters.

### Lordiphosa tripartita

Taxon classificationAnimaliaDipteraDrosophilidae

(Okada)

Drosophila (Hirtodrosophila) tripartita Okada, 1966: 78.
Lordiphosa tripartita : Zhang, 1993b: 145; Upadhyay and Singh 2007: 185.

#### Distribution.

Nepal, India (Uttarakhand).

### Lordiphosa nigricolor

Taxon classificationAnimaliaDipteraDrosophilidae

species group

Drosophila nigricolor species group, Laštovka & Máca, 1978: 418.
Lordiphosa nigricolor species group: Bächli et al., 2004: 264.

#### Diagnosis.

Acrostichal setulae in line with and anterior to dorsocentrals as long as others. Male foreleg tarsomeres without sex combs. Epandrium more or less projected or expanded on caudosubmedial margin near surstylus (Figs 3D, 4C, D, 6A, B, 7B).

#### Key to Indian species of the *nigricolor* group

**Table:** 

1	Epandrium with large flap covering surstylus on caudosubapical margin (Figs 4C, D, 6A, B); oviscapt medially broad and humped in lateral view, distally narrowing and curved ventrad, with large, sclerotized perineal plate between them (Figs 4G, H, 6E, F)	**2**
–	Epandrium without large flap on caudosubapical margin (Figs 3D, 7B); oviscapt neither medially humped in lateral view nor distally curved ventrad, without sclerotized perineal plate between them (Fig. 3G)	**3**
2.	Epandrial, caudosubapical flap serrate on dorsal margin (Fig. 4C, D)	***ayarpathaensis* Kandpal & Singh, sp. n.**
–	Epandrial, caudosubapical flap not serrate on dorsal margin (Fig. 6A, B)	***makaibarensis* Pradhan & Chatterjee, sp. n.**
3	Paramere apically not hirsute, unevenly bifurcated (Fig. 3F)	***antillaria* (Okada, 1984)**
–	Paramere apically hirsute (Fig. 7C, D)	**4**
4	Hypandrium not shorter than twice of width (Fig. 7C)	**5**
–	Hypandrium shorter than twice of width	**6**
5	Ventral branches of parameres symmetric in length, apically truncated; arista with five dorsal and two ventral branches in addition to terminal fork	***nigrovesca* (Lin & Ting, 1971)**
–	Ventral branches of parameres asymmetric in length, apically pointed (Fig. 7C, D); arista with 6–7 dorsal and 3–4 ventral branches (Fig. 7A)	***srinagarensis* Sati & Fartyal, sp. n.**
6	Epandrial, ventral lobe not extending beyond distal end of surstylus	***coei* (Okada, 1966)**
–	Epandrial, ventral lobe much elongated, extending beyond distal end of surstylus	**7**
7.	Hypandrium triangular, anteriorly narrowing; dm-cu crossvein somewhat clouded	***himalayana* (Gupta & Gupta, 1991)**
–	Hypandrium quadrate; dm-cu crossvein clear	***peniglobosa* (Kumar & Gupta, 1990)**

### Lordiphosa antillaria

Taxon classificationAnimaliaDipteraDrosophilidae

(Okada)

Fig. 3

Drosophila (Lordiphosa) antillaria Okada, 1984: 565.
Lordiphosa antillaria : Zhang et al., 1996: 349.
Drosophila (Lordiphosa) parantillaria Kumar & Gupta, 1990: 27. **Syn. n.**

#### Specimens examined.

INDIA: 5♂, 3♀, West Bengal, Assam, Bagdogra, 29 November 1981 (NSMT: National Museum of Nature and Science, Tsukuba, Japan; SEHU); 7♂, 1♀, Uttarakhand, Srinagar-Pauri Garhwal, Develgarh, 26 January 2011 (DZHNBGU, MZSIK, SEHU). MYNMAR: 1♂, Pyin Oo Lwin, 30 December 1981 (SEHU). TAIWAN: 1♂, Chitou, 20 January 1982 (SEHU); 11♂, 10♀, Chitou, 8 January 2008 (SEHU); 1♂, Fushan, 17 April 1997 (SEHU).

#### Diagnosis.

Paramere apically unevenly bifurcated (Fig. 3F). Aedeagus membranous, with numerous spinules (Fig. 3E).

#### Description

(supplementary and revised). **Adult male.** *Head*. Eye with sparse, interfacetal setulae. Occiput dark brown in upper half, pale yellow in lower half. Approximately 13–18 supracervical setae thin, apically more or less curved and pointed; postocular setae 12–18; occipital setae 7–11, including medial tiny ones. Dorsolateral arms of tentorial apodeme divergent, apically curved outward, reaching to fronto-orbital plate; dorsomedial arm half as long as dorsolateral arm. Interspace between antennal sockets narrower than half of socket width; first flagellomere grey, fringed with sparse, somewhat curved and long hairs on distal, outer margin, with only one small invaginated pouch; arista with 5–7 dorsal and 3–4 ventral branches in addition to terminal fork (Fig. 3B). Facial carina only slightly elevated, without setulae below. Subvibrissal seta distinctly shorter than vibrissa; additional row of oral setulae present above marginal row on anterior portion. Palpus with one prominent terminal and 3–4 short, subapical to lateroventral setae, without setulae on basal lobe (Fig. 3C). Cibarium thickened on anterior margin, not dilated laterad in anterior portion; anterolateral corners almost not projected; dorsal sclerite pear-shaped in dorsal view, anteriorly convex in lateral view; anterior sensilla two pairs, widely arranged in square behind anterior margin of hypopharynx; 28–37 medial sensilla arranged in anteriorly convergent rows; sensilla campaniformia two; posterior sensilla long, trichoid, anteriad curved, 20–22 arranged in anteriorly slightly convergent rows; somewhat sclerotized, thickened (in lateral view), anterior portion of hypopharynx 1/4 as long as cibarium (Fig. 3C). Labellum with five pseudotracheae (Fig. 3C).

*Thorax*. Posterior dorsocentral seta nearly equidistant from anterior margin of scutellum and anterior dorsocentral seta. Prescutellar setae absent. Anterior katepisternal seta as thin as aristal branches; no setula present anteriorly to anterior katepisternal seta.

*Wing*. Veins grey yellow; crossveins clear; bm-cu crossvein absent (Fig. 3A). C_1_ setae two, unequal in size.

*Legs*. Foreleg femur with approximately nine long setae in two rows on outer side; tarsus with neither tuft of dense, soft hairs on ventral side nor long setae.

*Abdomen*. Sternites pale grey; V and VI darker; VI setigerous.

*Terminalia* (Fig. 3D–F). Epandrium smoothly curved on posterior mid-dorsal margin, folded inward on ventral margin, pubescent except anterolateral margin and ventral lobe, triangularly pointed at insertion of surstylus (Fig. 3D). Surstylus articulated to epandrium, somewhat semicircular plate with neither pubescence nor trichoid setae on outer surface; prensisetae apically blunt (Fig. 3D). Cercus separated from epandrium, more or less sclerotized along anterior margin, nearly entirely pubescent except for posterior margin (Fig. 3D). Membrane between cercus and epandrium not pubescent (Fig. 3D). Lateral lobe of tenth sternite smaller than median lobe. Hypandrium dark brown, pubescent on small patches near caudolateral corners, approximately 1.5 times as long as wide, with a pair of inward extended plates apically articulated to ventral apices of parameres (Fig. 3E). Paramere distally curved posteriad, with 3–5 tiny sensilla in a row on proximal portion (Fig. 3F). Aedeagal basal process sclerotized, small, half as short as aedeagus, posteriorly connected through arch-shaped membrane bearing numerous tiny spinules to gonopod (Fig. 3F). Gonopods fused, forming somewhat semicircular plate (Fig. 3E).

*Measurements* (range in 6♂, in mm). BL = 1.40−1.72, ThL = 0.65−0.75, WL = 1.74−1.98, WW = 0.65−0.81.

*Indices* (range in 6♂, in ratio). FW/HW = 0.53−0.61, ch/o = 0.11−0.25, prorb = 0.40−0.73, rcorb = 0.07−0.27, vb = 0.30−0.63, dcl = 0.67−0.80, sctl = 1.36−1.54, sterno = 0.38−0.50, sterno2 (mid katepisternal seta / posterior katepisternal seta in length) = 0.10−0.33, orbito = 0.60−0.75, dcp = 0.25−0.43, sctlp = 1.27−1.33, C = 2.64−3.08, 4c = 0.80−1.00, 4v = 1.57−2.00, 5x = 1.40−1.88, ac = 2.40−3.25, M = 0.47−0.54, C3F = 0.27−0.45.

**Adult female.** Head, thorax, wings, and legs as in male.

*Terminalia* (Fig. 3G, H). Tergite VIII dark brown, ventrally broadened, pubescent only on caudodorsal margin, with 2−3 small setae near ventral margin (Fig. 3G). Epiproct and hypoproct pale greyish yellow; nearly entirely pubescent and setigerous (Fig. 3G). Oviscapt with approximately 12 marginal ovisensilla (proximal most and dorsal two trichoid but the others peg-like), approximately four lateral trichoid ones, ventro-subterminal trichoid one as long as dorsal marginal ones and approximately three apical small setae (Fig. 3G). Spermathecal capsule dark brown, spherical, smooth; introvert very shallow, 1/10 as deep as capsule height (Fig. 3H).

*Measurements* (1♀, in mm). BL = 1.79, ThL = 0.81, WL = 2.11, WW = 0.81.

*Indices* (1♀, in ratio). FW/HW = 0.56, ch/o = 0.11, prorb = 0.50, rcorb = 0.17, vb = 0.60, dcl = 0.56, sctl = 1.00, sterno = 0.63, sterno2 = 0.25, orbito = 0.75, dcp = 0.50, sctlp = 1.33, C = 3.00, 4c = 0.81, 4v = 1.75, 5x = 1.33, ac = 4.33, M = 0.50, C3F = 0.31.

#### Distribution.

Taiwan, China (Guangdong), Myanmar*, India (Sikkim, West Bengal*, Uttarakhand*) [* new record].

#### Remarks.

Kumar and Gupta (1990) described *L. parantillaria* (under the subgenus Lordiphosa of the genus *Drosophila*), based on 3♂ and 1♀ specimens collected from Ranipool, Gangtok district, Sikkim, India, distinguishing it from *L. antillaria* by the following characters: “arista branches 6/2 (6/5 in *antillaria*), 5X-index 1.6 (2.5 in *antillaria*) and anterior gonapophysis with 3 sensilla on basal half (with 9−10 sensilla on entire margin in *antillaria*)”. However, examining the specimens collected from Bagdogra, West Bengal, approximately 60 km south of Ranipool, in comparison with the specimens from Chitou, Taiwan (the type locality of *L. antillaria*), we found no significant differences in these and other characters between them (Fig. 3, Suppl. material 1). Thus, we here synonymize *Lordiphosa parantillaria* (Kumar & Gupta, 1990) with *Lordiphosa antillaria* (Okada, 1984). This species closely resembles *Lordiphosa subantillaria* (Okada, 1984) from Java and *Lordiphosa ramipara* (Zhang & Liang, 1992) in having the distally posteriad curved and bifurcated paramere, but can be distinguished from them by the diagnostic characters: in *subantillaria*, the paramere has short branch submedially and the aedeagus lacks spinules (Okada 1984: “Fig. 4”); in *ramipara*, the paramere has the longer branches equal in size (Zhang and Liang 1992: “Figs 2, 3”).

### Lordiphosa coei

Taxon classificationAnimaliaDipteraDrosophilidae

(Okada)

Drosophila (Sophophora) coei Okada, 1966: 82; Dwivedi and Gupta 1980: 88.
Lordiphosa coei : Wheeler, 1981: 54; Zhang et al. 1996: 349.
Drosophila (Sophophora) angusi Okada, 1977: 369.

#### Specimens examined.

CHINA: 8♂, 11♀, Sichuan, Mt. Emei, 550 m a.s.l., 16 July 1992; 14♂, 12♀, same data, except for 18 July 1992, 700–800 m a.s.l.; 3♂, 1♀, Hubei, Shennongjia, 26 July 1992 (SEHU).

#### Distribution.

China (Hubei*, Sichuan*, Guangdong), Nepal, India (West Bengal) [* new record].

### Lordiphosa himalayana

Taxon classificationAnimaliaDipteraDrosophilidae

(Gupta & Gupta)

Drosophila (Lordiphosa) himalayana Gupta & Gupta, 1991: 123.

#### Distribution.

India (Sikkim).

### Lordiphosa nigrovesca

Taxon classificationAnimaliaDipteraDrosophilidae

(Lin & Ting)

Drosophila (Phloridosa) nigrovesca Lin & Ting, 1971: 25 (as *nigrovescum*).
Lordiphosa nigrovesca : Zhang et al., 1996: 352.
Drosophila (Lordiphosa) aurantifrons Okada, 1984: 568.
Lordiphosa aurantifrons : De & Gupta, 1996: 131.

#### Distribution.

Taiwan, India (West Bengal).

### Lordiphosa ayarpathaensis

Taxon classificationAnimaliaDipteraDrosophilidae

Kandpal & Singh sp. n.

http://zoobank.org/E1222A9A-7A33-42E0-A456-2840AEE17747

Fig. 4

#### Type material.

*Holotype*. ♂: INDIA: Uttarakhand, Kumaon, Nainital district, Ayarpatha, 29°23'N, 79°27'E, 2,278 m a.s.l., 20−23 August 2009, M. C. Kandpal leg. (DZHNBGU).

*Paratypes*. INDIA: 5♂, 5♀, same data as the holotype; 10♂, 6♀, same data as the holotype except 3−5 September 2010 (DZHNBGU, SEHU).

#### Diagnosis.

Epandrium caudosubapically with large flap pointed apically, serrate on dorsal margin and covering largely surstylus (Fig. 4C, D). Paramere broader than aedeagal basal process, apically truncate, serrated; narrow, inward recurved, apically slightly pubescent elongation present at dorsal corner; sensilla 3–5, spaced in a longitudinal row (Fig. 4F).

#### Description.

**Adult male.** *Head*. Eye dark red, with sparse, interfacetal setulae. Supracervical setae 15–18, thin, apically more or less curved and pointed; postocular setae 16–19; occipital setae approximately 18, including medial tiny ones. Dorsolateral arms of tentorial apodeme divergent, nearly straight, reaching to fronto-orbital plate; dorsomedial arm half as long as dorsolateral arm. Occiput, ocellar triangle and fronto-orbital plates black; frontal vittae mat, light orange. Interspace between antennal sockets narrower than half of socket width; pedicel yellowish brown; first flagellomere grey, with only one small invaginated pouch; arista with 3–4 dorsal and two ventral branches in addition to terminal fork. Facial carina slightly elevated, narrower and shorter than first flagellomere, without setulae below. Gena and clypeus light brown. Subvibrissal seta distinctly shorter than vibrissa; additional row of oral setulae present above marginal row on anterior portion. Palpus with one prominent terminal and several short, subapical to lateroventral setae, without setulae on basal lobe. Cibarium thickened on anterior margin, not dilated laterad in anterior portion; anterolateral corners almost not projected; dorsal sclerite pear-shaped in dorsal view, anteriorly convex in lateral view; anterior sensilla two pairs, widely arranged in square behind anterior margin of hypopharynx; 32–33 medial sensilla arranged in mostly parallel but anteriorly convergent rows; sensilla campaniformia two; posterior sensilla very long, trichoid, gently curved forward, approximately 22, arranged in anteriorly divergent rows; somewhat sclerotized, thickened (in lateral view), anterior portion of hypopharynx shorter than 1/5 length of cibarium. Prementum slightly thicker in ventral than in dorsal portion from lateral view (Fig. 4A), nearly parallel-sided in posterior view (Fig. 4B). Labellum with five pseudotracheae per side (Fig. 4A).

*Thorax*. Postpronotal lobe grey yellow, with two prominent setae: lower one longer. Scutum and scutellum glossy, light brown. Thoracic pleura greyish brown. Posterior dorsocentral seta situated nearer to anterior dorsocentral seta than to anterior margin of scutellum. Prescutellar setae absent. Acrostichal setulae in six rows. Basal scutellar setae parallel or convergent; apicals cruciate. Anterior katepisternal seta thicker than aristal branches; no setula present anteriorly to anterior katepisternal seta.

*Wing* hyaline. Veins light brown; crossveins not clouded; bm-cu crossvein absent; R_2+3_ nearly straight; R_4+5_ and M_1_ nearly parallel. C_1_ setae two, unequal in size. Halter opaque white.

*Legs* light brown; last two tarsomeres of all legs darker. Foreleg femur with approximately eight long setae in two rows on ventral and outer surfaces; tarsus without any sexual ornamentation. Foreleg tarsomere I as long as three succeeding tarsomeres together; mid-leg one slightly longer than three succeeding tarsomeres together; hindleg one slightly shorter than rest together. Preapical, dorsal setae present on tibiae of all legs; apical setae on tibiae of fore- and mid-legs.

*Abdomen*. Tergites I to IV medially, widely yellow, laterally brown; V and VI nearly entirely dark brown; each tergite with small setae in approximately three rows and large setae on posterior margin. Sternites light brown; setigerous VI present.

*Terminalia* (Fig. 4C–F). Epandrium smoothly curved on posterior mid-dorsal margin, folded inward on caudoventral margin, nearly entirely pubescent except anterolateral margin, lower portion of ventral lobe and apical portion of caudo-subapical flap, with approximately seven setae on medial to dorsal portion, 10–11 setae on ventral lobe and sclerotized process at caudoventral apex (Fig. 4C, D). Surstylus articulated to epandrium, distally narrowing and apically truncate, pubescent medially on outer surface; distal margin with a row of 17–18 stout, trichoid prensisetae on dorsal portion and two or three irregular rows of such setae on ventral portion; outer surface lacking peg-like seta.(Fig. 4C, D). Cercus separated from epandrium, nearly entirely pubescent, with 24–25 setae medially to dorsally, ventro-apically truncate and with 3–4 prominent, curved setae on margin and small, apically round projection at anterior corner (Fig. 4C, D). Membrane between cercus and epandrium not pubescent (Fig. 4C). Lateral lobe of tenth sternite smaller than median lobe. Hypandrium anteriorly narrowing, with a pair of narrow plates connecting between bases of parameres and lateral margins of hypandrium (Fig. 4E). Paramere very long, sclerotized process, articulated to basal part of aedeagus, basally much elongated and curved like hook; basal elongation apically pointed, subapically articulated to tip of hypandrial, lateral plate (Fig. 4F). Aedeagus membranous, apically shaped like “funnel” with hirsute margin, apicodorsally connected with gonopod by membrane, basally fused to apodeme; basal process strongly sclerotized, slightly shorter than aedeagus, apically hamate and pointed; apodeme rod-like, as long as aedeagus (Fig. 4E, F). Gonopods fused, forming plate situated dorsally to aedeagus (Fig. 4E, F).

*Measurements* (holotype / range in 6♂ paratypes, in mm). BL = 2.59 / 2.04−2.41, ThL = 1.22 / 0.96−1.11, WL = 2.96 /2.48−2.96, WW = 1.15 / 0.85−1.26.

*Indices* (holotype / range in 6♂ paratypes, in ratio). FW/HW = 0.50 / 0.44−0.67, ch/o = 0.11 / 0.10−0.22, prorb = 0.60 / 0.50−0.89, rcorb = 0.20 / 0.17−0.40, vb = 0.40 / 0.50−0.67, dcl = 0.55 / 0.36−0.60, sctl = 1.33 / 1.18−1.56, sterno = 0.33 / 0.25−0.40, sterno2 = 0.22 / 0.13−0.33, orbito = 1.00 / 0.50−1.00, dcp = 0.44 / 0.30−0.57, sctlp = 1.20 / 1.20−1.33, C =3.64 /2.87−3.33, 4c = 0.67 / 0.67−0.75, 4v = 1.67 / 1.14−1.55, 5x = 1.43 / 1.17−1.67, ac = 2.00 / 2.14−2.50, M = 0.48 / 0.37−0.48, C3F = 0.23 / 0.11−0.21.

**Adult female.** Head, thorax, wings, and legs as in male.

*Terminalia* (Fig. 4G−I). Tergite VIII short, ventrally tapering, without setae, pubescent only on dorsocaudal portion (Fig. 4G). Oviscapt medially broad and humped in lateral view, distally narrowing and curved ventrad, with three stout, peg-like ovisensilla and ventro-subterminal, trichoid one on apical margin, and approximately six trichoid, lateral ones on distal surface (Fig. 4G). Large, sclerotized perineal plate present between oviscapts (Fig. 4G, H). Spermathecal capsule elongated dome-shaped, with horizontal wrinkles basally and somewhat irregular ones on apical surface; introvert half as deep as capsule height (Fig. 4I).

*Measurements* (range in 5♀ paratypes, in mm). BL = 2.22−2.52, ThL = 1.04−1.41, WL = 2.85−3.15, WW = 1.04−1.37.

*Indices* (range in 5♀ paratypes, in ratio). FW/HW = 0.50−0.65, ch/o = 0.10−0.30, prorb = 0.50−0.70, rcorb = 0.17−0.33, vb = 0.33−0.67, dcl = 0.46−0.67, sctl = 1.20−1.36, sterno = 0.25−0.60, sterno2 = 0.13−0.33, orbito = 0.67−1.33, dcp = 0.40−0.50, sctlp = 0.80−1.25, C = 3.13−3.85, 4c = 0.60−0.67, 4v = 1.38−1.67, 5x = 1.17−1.80, ac = 2.17−2.67, M = 0.33−0.45, C3F = 0.20−0.27.

#### Etymology.

Pertaining to type locality.

#### Distribution.

India (Uttarakhand).

#### Flower visitation.

**Figure 5.**
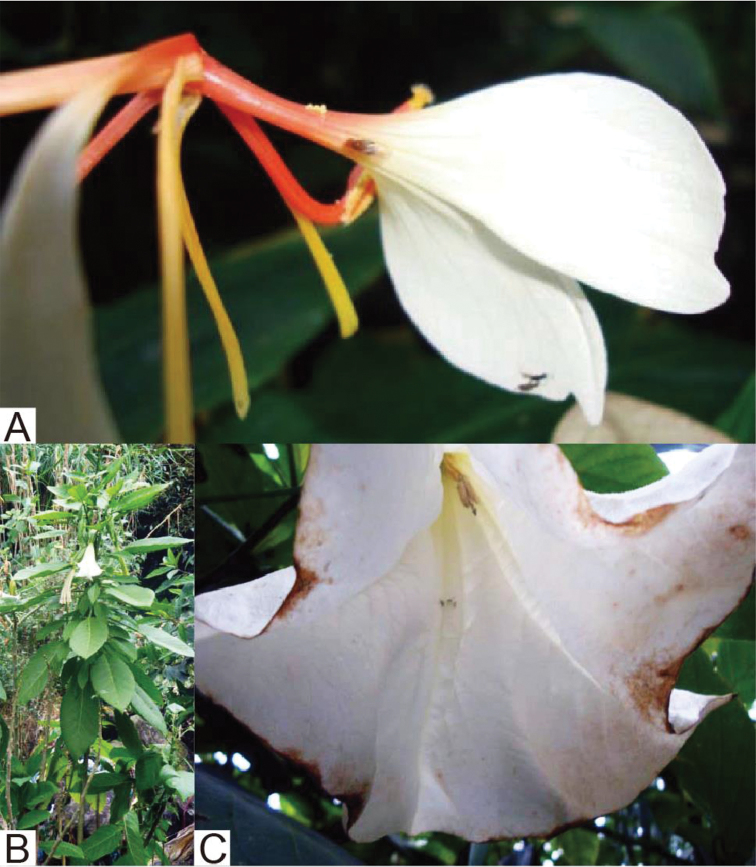
Flowers visited by *Lordiphosa* flies: **A** *Hedychium spicatum* (Zingiberaceae) visited by *L. ayarpathaensis* sp. n. **B, C** *Datura suaveolens* (Solanaceae) visited by *L. makaibarensis* sp. n.

Adult flies were collected from flowers of *Hedychium spicatum* (local name: Haldu, Kapur Kachri or Sand harlika; English common name: Spiked Ginger Lily; Fig. 5A), a smallish, hardy, perennial herb, belonging to the family Zingiberaceae, with fleshy rhizomes, green, broadly lanceolate leaves, straight stem (up to approximately 1 m high) and large orange and white flowers. It grows throughout subtropical Himalaya in the Indian states of Assam, Arunachal Pradesh and Uttarakhand, with an altitudinal range of 1,000 m to 3,000 m.

#### Remarks.

**Figure 6.**
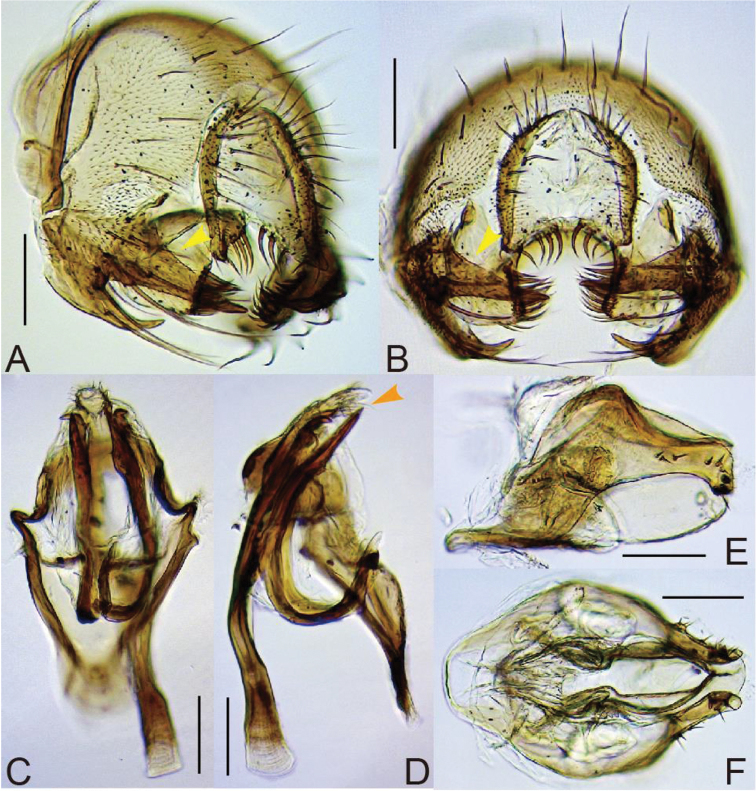
*Lordiphosa makaibarensis* Pradhan & Chatterjee, sp. n. (♂ paratype from Kurseong, Darjeeling, West Bengal, India): **A, B** periphallic organs (**A** caudolateral view **B** caudoventral view) **C, D** phallic organs (**C** ventral view **D** lateral view) **E, F** oviscapt (**E** lateral view **F** ventral view). Scale bars 0.1 mm.

**Figure 7.**
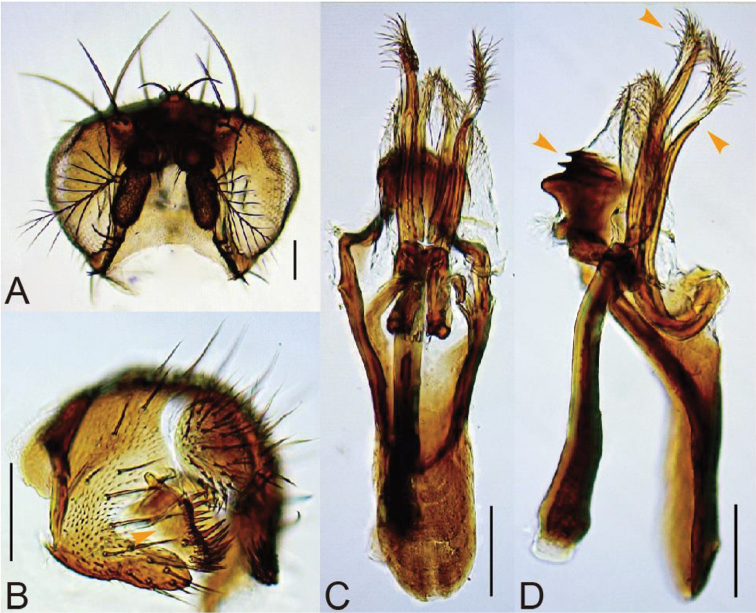
*Lordiphosa srinagarensis* Sati & Fartyal, sp. n. (♂ paratype from HNB Garhwal University, Srinagar, Garhwal, Uttarakhand, India): **A** head (frontal view) **B** periphallic organs (caudolateral view) **C, D** phallic organs (**C** ventral view **D** lateral view). Scale bars 0.1 mm.

This species somewhat resembles *L. nigrovesca* in having the sclerotized, apically pointed process at caudoventral apex of epandrium and the paramere apically truncated, but differs from it in the color of thorax and abdomen (glossy black in *nigrovesca*), the caudosubapical, large flap of epandrium (absent; Okada 1984: “Fig. 8”, De and Gupta 1996: “Fig. 6”), the position of apically pubescent process on the paramere (subapical; Okada 1984: “Fig. 9”, De and Gupta 1996: “Fig. 7”), the shape of oviscapt (distally broad; De and Gupta 1996: “Fig. 8”) and the number of ovisensilla (11 marginals and six laterals; De and Gupta 1996: “Fig. 8”). Molecular data of this species are available from Sarswat et al. (2016).

### Lordiphosa makaibarensis

Taxon classificationAnimaliaDipteraDrosophilidae

Pradhan & Chatterjee sp. n.

http://zoobank.org/794AE213-2170-4321-A9D1-DD3B65119051

Fig. 6

#### Type material.

*Holotype*. ♂: INDIA: West Bengal, Darjeeling, Kurseong, 26°53'N, 88°17'E, 1,458 m a.s.l., 1 September 2010, S. Pradhan leg. (DZHNBGU).

*Paratypes*. INDIA: 5♂, 5♀, same data as the holotype (DZHNBGU, SEHU).

#### Diagnosis.

Epandrial, caudosubapical, large flap not serrate on dorsal margin (Fig. 6A, B). Paramere as broad as aedeagal basal process, apically pointed, sclerotized and without pubescence, subapically not serrate; sensilla approximately four, arranged relatively compactly in an irregular row on submedial portion (Fig. 6C, D).

#### Description

(not referring to characters commonly seen in the foregoing species, *L. ayarpathaensis*). **Adult male.** *Head*. Supracervical setae 10–15; postocular setae 12–13; occipital setae 11–13. Occiput, ocellar triangle and fronto-orbital plates brownish yellow. Antennal pedicel light brownish yellow; first flagellomere light grey; arista with 3–4 dorsal and 1–2 ventral branches in addition to terminal fork. Gena and clypeus brownish yellow. Cibarial medial sensilla 22–23; posterior sensilla approximately 16.

*Thorax* light brownish yellow. Posterior dorsocentral seta situated nearer to anterior dorsocentral seta than to anterior margin of scutellum.

*Wing*. Veins greyish yellow.

*Legs* light brownish yellow. Foreleg femur with approximately five long setae in two rows on ventral and outer surfaces.

*Abdomen*. Tergites nearly entirely yellow; each tergite with small setae in approximately two rows and large setae on posterior margin. Sternites off-white.

*Terminalia* (Fig. 6A–D). Epandrium with 6–9 setae on medial to dorsal portion and approximately nine setae on ventral lobe (Fig. 6A). Surstylus with approximately 15 stout, trichoid prensisetae in a single row dorsally but in two or three irregular rows ventrally on distal margin (Fig. 6B). Cercus with 16–17 setae medially to dorsally, ventro-apically truncate and with approximately four prominent, curved setae on margin and small, apically somewhat pointed projection at anterior corner (Fig. 6A, B).

*Measurements* (holotype / range in 5♂ paratypes, in mm). BL = 1.78 / 1. 48–1.81, ThL = 0.81 / 0.74–0.85, WL = 2.22 / 2.22–2.44, WW = 0.74 / 0.81–0.85.

*Indices* (holotype / range in 3♂ paratypes, in ratio). FW/HW = 0.50 / 0.44–0.53, ch/o = 0.13 / 0.10–0.31, prorb = 0.83 / 0.63–0.78, rcorb = 0.17 / 0.22–0.44, vb = 0.40 / 0.31–0.50, dcl = 0.61 / 0.56–0.71, sctl = 1.38 / 1.17–1.35, sterno = 0.25 / 0.30–0.50, sterno2 = 0.13 / 0.10−0.38, orbito = 0.25 / 0.48–0.80, dcp = 0.57 / 0.47–0.62, sctlp = 1.00 / 0.75–0.97, C = 3.40 / 2.91–3.18, 4c = 0.77 / 0.67–0.79, 4v = 1.69 / 1.39–1.69, 5x = 1.00 / 1.10–1.75, ac = 2.00 / 2.00–2.75, M = 0.31 / 0.34–0.47, C3F = 0.30 / 0.17–0.27.

**Adult female.** *Terminalia*. Oviscapt with approximately five trichoid, lateral ovisensilla (Fig. 6E, F).

#### Etymology.

Partaining to “Makaibari tea estates”. Makaibari was the first tea factory in the world, established in 1859, in Kurseong, Darjeeling, West Bengal.

#### Distribution.

India (West Bengal).

#### Flower visitation.

Adult flies of this species were collected from flowers of *Datura suaveolens* (local name: Dhokrey; English common name: Angel trumpet or Devils trumpet; Fig. 5B, C), an exotic plant belonging to the Solanaceae. It was introduced from South America and is now found growing along riverbeds or forest edges at moist places almost all over India.

#### Remarks.

This species closely resembles the foregoing species, *L. ayarpathaensis*, in having the large flap on caudosubapical margin of epandrium, the oviscapt medially broad and humped in lateral view and distally narrowing and curved ventrad, and the large, sclerotized perineal plate present between oviscapts, but can be distinguished from it by the smaller size and paler color of the body and the diagnostic characters.

### Lordiphosa peniglobosa

Taxon classificationAnimaliaDipteraDrosophilidae

(Kumar & Gupta)

Drosophila (Lordiphosa) peniglobosa Kumar & Gupta, 1990: 25.

#### Distribution.

India (West Bengal).

### Lordiphosa srinagarensis

Taxon classificationAnimaliaDipteraDrosophilidae

Sati & Fartyal sp. n.

http://zoobank.org/117C76F6-4F6B-44FF-9659-6D94DB4C19E0

Fig. 7

#### Type material.

*Holotype*. ♂: INDIA: Uttarakhand, Srinagar Garhwal, Tehri district, HNBGU Forestry Nursery Chauras Campus, 30°13'N, 78°47'E, 560 m a.s.l., 16 December 2010, R. S. Fartyal leg. (DZHNBGU).

*Paratypes*. INDIA: 3♂, same data as the holotype (DZHNBGU, MZSIK, SEHU).

#### Diagnosis.

Surstylus with neither pubescence nor stout setae on outer surface (Fig. 7B). Paramere distally bifurcated into ventral, sclerotized, apically pointed branch and dorsal, less sclerotized, apically hirsute branch; ventral branches asymmetric in length: left one longer (Fig. 7C, D). Gonopods with medial broad and two apical narrow ridges in lateral view (Fig. 7D).

#### Description.

**Adult male.** *Head*. Eye red, with sparse interfacetal setulae (Fig. 7A). Occiput glossy, dark brown in dorsal half, grey yellow in ventral half. Supracervical setae 14–19, thin, apically more or less curved and pointed; postocular setae 14–16; occipital setae 12–13, including tiny medial ones. Dorsolateral arms of tentorial apodeme divergent, apically curved outwards, reaching to fronto-orbital plate; dorsomedial arm half as long as dorsolateral arm. Frons grey yellow except for dark brown upper portion of fronto-orbital plate and medial portion of ocellar triangle. Interspace between antennal sockets narrower than half of socket width; pedicel grey brown; first flagellomere grey, fringed with sparse, somewhat curved and long hairs on distal, outer margin, with only one small invaginated pouch; arista with 6–7 dorsal and 3–4 ventral branches in addition to terminal fork (Fig. 7A). Face grey-yellow; carina only slightly elevated, without setulae below (Fig. 7A). Gena grey-yellow, with dark brown, medial patch and ventral margin; subvibrissal seta distinctly shorter than vibrissa; additional row of oral setulae present above marginal row on anterior portion. Clypeus grey-brown. Palpus dark grey, with one prominent terminal and several short subapical to lateromedian setae, without setulae on basal lobe. Cibarium thickened on anterior margin, not dilated laterad in anterior portion; anterolateral corners almost not projected; dorsal sclerite pear-shaped in dorsal view, anteriorly convex in lateral view; anterior sensilla two pairs, widely arranged in square behind anterior margin of hypopharynx; 24–29 medial sensilla arranged in anteriorly convergent rows; sensilla campaniformia two; posterior sensilla long, trichoid, nearly straight, approximately 27, arranged in nearly parallel rows; somewhat sclerotized, thickened (in lateral view) anterior portion of hypopharynx 1/4 as long as cibarium. Labellum with five pseudotracheae.

*Thorax.* Nearly entirely brownish black. Posterior dorsocentral seta nearly equidistant from anterior margin of scutellum and anterior dorsocentral seta. Prescutellar setae absent. Acrostichal setulae in six rows. Basal scutellar setae parallel; apicals cruciate. Anterior katepisternal seta as thin as aristal branches; no setula present anteriorly to anterior katepisternal seta.

*Wing* slightly fuscous; veins grey brown; cross veins clear; bm-cu crossvein absent; R_2+3_ nearly straight; R_4+5_ and M_1_ nearly parallel. C_1_ setae two, unequal in size. Halter opaque white.

*Legs* grey yellow. Foreleg femur with approximately six long setae in two rows on outer side; tarsus without any sexual ornamentation. Foreleg tarsomere I as long as three succeeding tarsomeres together; mid- and hind-leg ones as long as rest together. Preapical, dorsal setae present on tibiae of all legs; apical setae on tibiae of fore- and mid-legs.

*Abdomen*. Tergites entirely glossy, brownish black, each with setae arranged in roughly four transverse rows: those in last row longest. Sternites pale to dark grey; setigerous VI present.

*Terminalia* (Fig. 7B–D). Epandrium brown, smoothly curved on posterior mid-dorsal margin, nearly entirely pubescent except anterolateral margin and caudosubmedial portion, expanded on caudosubmedial margin at insertion of surstylus, caudoventrally developed into ventral lobe extended posteriad and apically slightly pointed, with about 5–7 setae on medial to dorsal portion, 9–12 setae on ventral lobe and unpubescent, inward fold on ventral margin (Fig. 7B). Surstylus articulated to epandrium, wide, somewhat triangular plate, with 9–10 apically pointed prensisetae reducing in size below in a row on distal margin and 13–16 recurved setae on caudoventral, inner portion (Fig. 7B). Cercus separated from epandrium, pubescent only medially, with 20–24 long setae medially to dorsally and 13–14 short ones on ventral portion (Fig. 7B). Membrane between cercus and epandrium unpubescent (Fig. 7B). Lateral lobe of tenth sternite larger than median lobe. Hypandrium dark brown, completely unpubescent, approximately thrice as long as wide, with a pair of inward extended plates apically articulated to ventral apices of parameres (Fig. 7C). Paramere longer than aedeagus, basally curved ventrad and U-shaped in lateral view, with approximately three minute sensilla in a row proximally (Fig. 7D). Aedeagal basal processes distally fused to membranous aedeagus and membrane posteriorly connected to gonopod; this composite distally hirsute (Fig. 7C, D). Gonopods fused with each other, forming roof-like plate (Fig. 7C, D).

*Measurements* (holotype / range in 2♂ paratypes, in mm). BL = 1.69 / 1.66−1.69, ThL = 0.65 / 0.65−0.75, WL = 2.05 / 1.95−2.05, WW = 1.04 / 0.81−1.04.

*Indices* (holotype / range in 2♂ paratypes, in ratio). FW/HW = 0.50 / 0.50−0.53, ch/o = 0.17 / 0.17−0.22, prorb = 0.71 / 0.57−0.71, rcorb = 0.33 / 0.33−0.57, vb = 0.50 / 0.50−0.63, dcl = 0.55 / 0.55−0.70, sctl = 1.50 / 1.44−1.50, sterno = 0.88 / 0.56−0.88, sterno2 = 0.50 / 0.44−0.50, orbito = 0.80 / 0.50−0.80, dcp = 0.43 / 0.43−0.50, sctlp = 0.63 / 0.63−0.80, C = 2.64 / 2.64−2.92, 4c = 0.88 / 0.88−0.92, 4v = 1.63 / 1.63−1.77, 5x = 1.60 / 1.33−1.60, ac =2.33 / 2.33−2.40, M = 0.50 / 0.50−0.62, C3F = 0.29 / 0.25−0.29.

#### Etymology.

Referring to the type locality.

#### Distribution.

India (Uttarakhand).

#### Remarks.

This species closely resembles *Lordiphosa penicilla* (Zhang, 1993) from southwestern China in the morphology of male terminalia, but can be distinguished from it by the diagnostic characters: in *L. penicilla*, surstylus medially pubescent and with several stout setae on outer surface; sclerotized ventral branches of parameres symmetric in length; gonopod with single medial ridge in lateral view (Zhang 1993a: “Figs 4–6”).

## Discussion

In this study, it was found that *L. neokurokawai* has a special type of sex comb composed of thick setae of approximately 15 TBRs along the entire length of tarsomere I of male foreleg (Fig. 1B), which was overlooked in its original description by Singh and Gupta (1981). This finding is important for considering the evolution of sex comb in the genus *Lordiphosa*. The sex comb is a male-specific morphological structure composed of thickened setae (“teeth”) that develops on the foreleg tarsus of adult male in the Drosophilidae. This male-specific character is seen only in *Sophophora* and *Lordiphosa* (Hu and Toda 2001), and is used variously in tactile interactions between males and females during courtship and mating behavior (Spieth 1952; see also Kopp 2011 for a review of sex comb functions). Likely in relation to its use as an important component of mating behavior, the sex comb varies in structure even between closely related species, implying that its rapid diversification would have been driven by sexual selection (Markow et al. 1996, Kopp 2011). Three major patterns are recognized in the sex comb structure: (i) “transverse” sex comb comprising TBR(s) of thickened setae on the distal portion of tarsomere; (ii) “oblique” one of row(s) more or less rotated and moderate in length on the distal portion of tarsomere; and (iii) “longitudinal” one aligned along the nearly entire length of tarsomere (Kopp and True 2002, Atallah et al. 2009). All four known species of the *Lordiphosa miki* species group have extended “longitudinal” sex combs of the last type (Laštovka and Máca 1978, Okada 1984, Kopp 2011). The phenotypically identical “longitudinal” sex combs are present in the *melanogaster* and *obscura* species groups of the subgenus Sophophora (Kopp 2011, Atallah et al. 2012), explaining why members of the *miki* group had once been assigned to the subgenus Sophophora (Kikkawa and Peng 1938, Okada 1956, Lee 1959, Bock and Wheeler 1972). Species of the *L. denticeps* group possess the “transverse” sex combs on the foreleg tarsomeres I to III (Kopp 2011, Atallah et al. 2012). However, probably because the sex comb teeth of the *denticeps* group are less prominent than those of *Sophophora*, this structure had been overlooked in earlier descriptions of *denticeps*-group species until Zhang (1993b) first recognized it. The two other species groups, i.e., *fenestrarum* and *nigricolor* ones, of *Lordiphosa* lack sex combs. Interestingly, the Neotropical *Sophophora* comprising the *saltans* and the *willistoni* groups, which is the sister clade of *Lordiphosa* (Gao et al. 2011), has no sex comb either. This character distribution pattern across *Lordiphosa* and *Sophophora* suggests two hypotheses for the evolution of sex comb. One is the “single-origin” hypothesis: the sex comb was acquired in the common ancestor of *Lordiphosa* and *Sophophora*, and secondarily lost in several lineages. The other is the “multiple-origin” hypothesis: the sex comb evolved independently on several lineages. To date, any approach from the phylogenetic analysis has not succeeded in distinguishing between these two hypotheses. Another possible way is to elucidate the real homology of similar phenotypes by studying the molecular processes underlying their development. Recent evo-devo studies have succeeded in revealing that similar phenotypic structures in sex comb result from different developmental mechanisms (Atallah et al. 2009, 2012, Tanaka et al. 2009, Kopp 2011). For instance, the “longitudinal” sex combs seen in the *melanogaster* and *obscura* groups develop, under similar regulation by the same key genes, through different cellular mechanisms. In some species, such as *Drosophila rhopaloa* Bock & Wheeler, 1972 of the *melanogaster* group and *Drosophila guanche* Monclus, 1976 of the *obscura* group, the “longitudinal” sex comb originates from one or a few distal, transverse rows of bristle-precursor cells that are homologous to those for female TBRs but subsequently rotate 90° and align to form a longitudinal row (Tanaka et al. 2009, Atallah et al. 2012). In species of the *montium* subgroup and *Drosophila ficusphila* Kikkawa & Peng, 1938 of the *melanogaster* group, however, the sex comb arises from male-specific precursor cells aligned in a longitudinal row on the presumptive region (Tanaka et al. 2009, Atallah et al. 2012). Furthermore, Atallah et al. (2012) found the third developmental mode of “longitudinal” sex comb in *Lordiphosa magnipectinata* (Okada, 1956) of the *miki* group: the sex comb development starts from the ancestral, sexually monomorphic arrangement of TBR precursor cells; then, most of such short, transverse rows of precursors rotate independently of each other and eventually assemble into a contiguous, longitudinal row. In relation to this developmental process of “longitudinal” sex comb in the *miki* group, the sex comb of *L. neokurokawai* of the *denticeps* group, which consists of multiple transverse combs arranged along the entire length of tarsomere I, may represent an intermediate stage, i.e., before rotation of TBRs, of the sex comb development in the *miki* group. Taken together these results support a common origin for sex combs in *Lordiphosa*.

## Supplementary Material

XML Treatment for Lordiphosa

XML Treatment for Lordiphosa denticeps

XML Treatment for Lordiphosa neokurokawai

XML Treatment for Lordiphosa curva

XML Treatment for Lordiphosa tripartita

XML Treatment for Lordiphosa nigricolor

XML Treatment for Lordiphosa antillaria

XML Treatment for Lordiphosa coei

XML Treatment for Lordiphosa himalayana

XML Treatment for Lordiphosa nigrovesca

XML Treatment for Lordiphosa ayarpathaensis

XML Treatment for Lordiphosa makaibarensis

XML Treatment for Lordiphosa peniglobosa

XML Treatment for Lordiphosa srinagarensis

## Supplementary materials

Supplementary material 1 Figure S1 Data type: multimedia Explanation note: *Lordiphosa antillaria* (Okada, 1984) (♂♀ from the type locality: Chitou, Taiwan). This dataset is made available under the Open Database License (http://opendatacommons.org/licenses/odbl/1.0/). The Open Database License (ODbL) is a license agreement intended to allow users to freely share, modify, and use this Dataset while maintaining this same freedom for others, provided that the original source and author(s) are credited.Rajendra S. Fartyal, Pradeep C. Sati, Sushmika Pradhan, Mukul C. Kandpal, Masanori J. Toda, Rabindra N. Chatterjee, Birendra K. Singh
