# Supplementary material for: A review of the genus Lordiphosa Basden in India, with descriptions of four new species from the Himalayan region (Diptera, Drosophilidae)
Source: Zookeys. 2017 Aug 8;(688):49–79. doi: 10.3897/zookeys.688.12590 (PMC5672582; doi:10.3897/zookeys.688.12590)
Supplement: Supplementary material 1 — Figure S1 [file zookeys-688-049-s001.pdf]

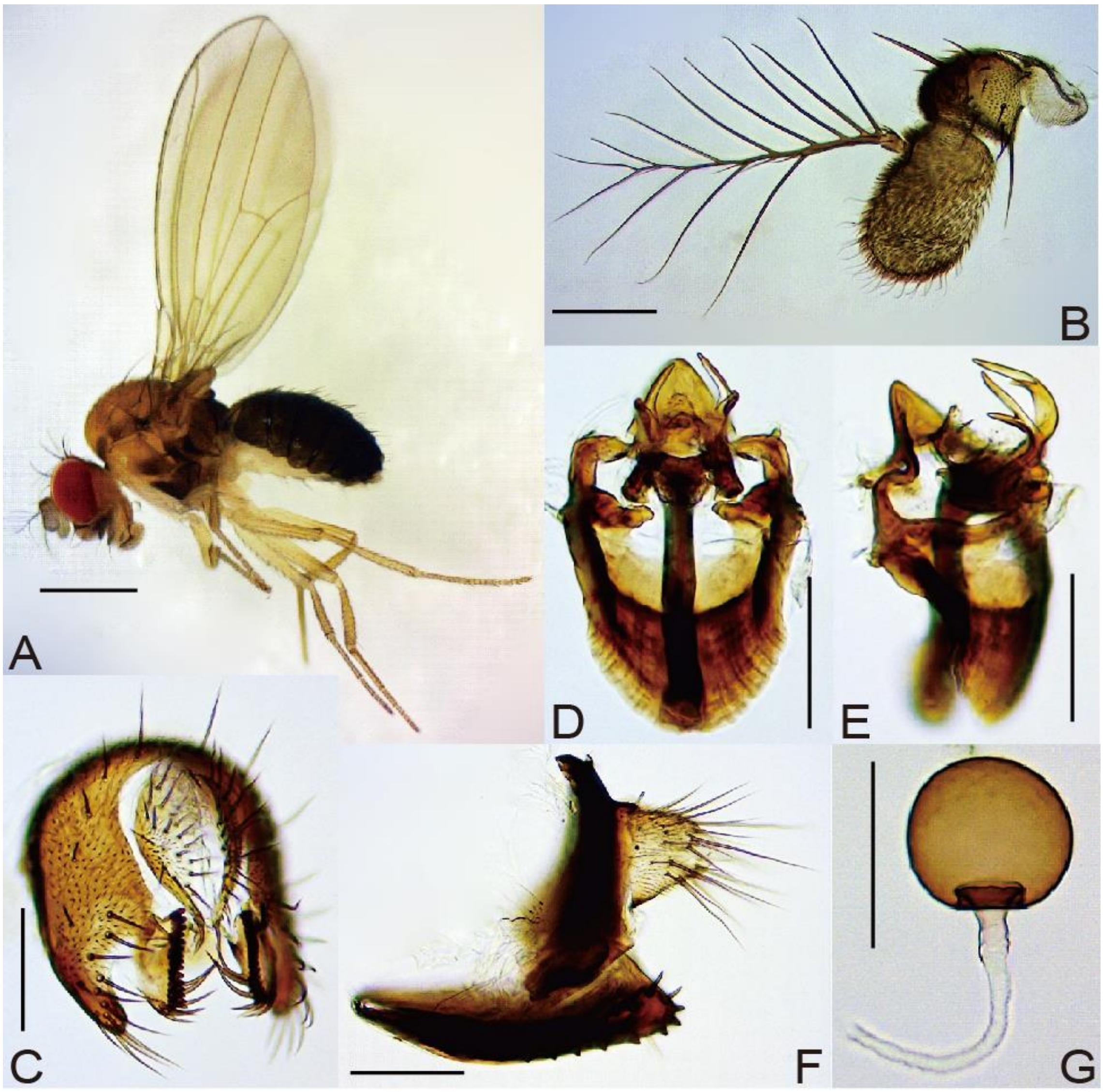

Figure S1 *Lordiphosa antillaria* (Okada, 1984) (♂♀ from the type locality: Chitou, Taiwan): (A) ♂ whole body; (B) antenna; (C) periphallic organs (caudolateral view); (D, E) phallic organs (D: ventral view, E: ventrolateral view); (F) ♀ terminalia; (G) spermatheca. Scale bars: 0.5 mm in A, 0.1 mm in B–G.
